# COMMUNITY-BASED GARDENING INTERVENTIONS FOR ASIAN AMERICAN IMMIGRANTS AND REFUGEES: A SCOPING REVIEW

**DOI:** 10.1093/geroni/igae098.4334

**Published:** 2024-12-31

**Authors:** Lan Doan, Casandra Chen, My Duyen Tran, Stella Yi, Simona Kwon

**Affiliations:** NYU Langone Health, NYU Grossman School of Medicine, New York, United States; NYU Grossman School of Medicine, New York, New York, United States; NYU Grossman School of Medicine, New York, New York, United States; NYU Grossman School of Medicine, New York, New York, United States; NYU Grossman School of Medicine, New York, New York, United States

## Abstract

Gardening and horticultural therapy have been shown to increase overall sense of a community, social engagement and quality of life, and increase physical activity. Gardening interventions are innovative means to address social isolation and loneliness by engaging adults in a meaningful way and building supportive communities that integrate informative and respectful interactions around stigmatized topics like social isolation and loneliness and culturally specific contexts and experiences way (e.g., combining gardening interests with culturally relevant foods). However, few interventions have been developed for diverse cultural communities with limited English ability and there are no known interventions targeting isolation and loneliness among older Asian American adults who face severe economic and language challenges that limit health access. This scoping review examines gardening-based interventions for Asian American populations, specifically older adults. Following PRISMA guidelines, we searched PubMed, CINAHL, PsycINFO, and Embase in June 2024 for peer-reviewed articles related to gardening, health interventions and Asian American people in the US. Of the 1419 studies identified, 25 were reviewed in full and 6 studies were included. Key themes include cultural tailoring of garden-based nutrition programs; physical and mental health benefits of community gardens for refugees and immigrants; and that role of community gardens in fostering community social support and individual sense of belonging. Studies focused on adults and older adults and Bhutanese, Karen, Nepali, and Southeast Asian communities. These findings highlight the limited studies available for Asian American communities and urgent need for developing culturally relevant healthy aging interventions for diverse refugee and immigrant populations.

